# Identifying risk factors for pregnancy and lactation-associated osteoporosis: insights from an Italian survey in PLAO patients and controls

**DOI:** 10.3389/fendo.2025.1612188

**Published:** 2025-07-22

**Authors:** Giorgia Grassi, Marta Zampogna, Anna Atlasova, Sara Rondinella, Alberto Ghielmetti, Giordana Siracusano, Iacopo Chiodini, Giovanna Mantovani, Cristina Eller-Vainicher

**Affiliations:** ^1^ Endocrine Unit, Fondazione IRCCS Cà Granda Ospedale Maggiore Policlinico, Milan, Italy; ^2^ Department of Clinical Sciences and Community Health, Dipartimento di Eccellenza 2023-2027, University of Milan, Milan, Italy; ^3^ Endocrine Unit, University Hospital G. Martino, Messina, Italy; ^4^ Department of Human Pathology, University of Messina, Messina, Italy; ^5^ Department of Medical Biotechnology and Translational Medicine, University of Milan, Milan, Italy; ^6^ Unit of Endocrinology, ASST Grande Ospedale Metropolitano Niguarda, Milan, Italy

**Keywords:** fracture, lactation, osteoporosis, pregnancy, fracture risk assessment

## Abstract

**Background:**

Pregnancy and lactation-associated osteoporosis (PLAO) is characterized by fragility fractures during late pregnancy and postpartum period. The risk factors for PLAO are largely unknown. This study aims to identify the risk factors for PLAO, related and unrelated to pregnancy.

**Patients and methods:**

A questionnaire on clinical history, lifestyle factors, nutritional habits, and pregnancy-related characteristics was administered to 155 women with PLAO and 182 women without PLAO.

**Results:**

Women with PLAO were older (32.9 ± 4.7 years), showed a higher prevalence of BMI <18.5 Kg/m2 (23.6%), eating disorders (11.6%), amenorrhea (7.1%), chronic diseases associated with bone fragility (11.7%), calcium-intake <500 mg/day (66.9%), previous fragility fractures (18.7%), blue sclerae (5.2%) and family history of vertebral/femoral FXs (21.0%) than women without PLAO (31.6 ± 5.4 years, 13.8%, 5.5%, 2.2%, 2.6%, 50.8%, 6.0%, 0.5%, 11.0%, p<0.05 for all comparisons). The risk of PLAO was associated with pre-pregnancy fragility fractures (odds ratio, OR 2.7, 95% Confidence Interval, CI, 1.199-6.036, p=0.016), chronic diseases (OR 5.8 95% CI, 1.919-17.562, p=0.002), age at pregnancy (OR 1.1, 95% CI, 1.005-1.109, p=0.030), BMI<18.5 Kg/m^2^ (OR 2.0, 95% CI, 1.042-3.902, p=0.037) and calcium-intake <500 mg/day (OR 2.5 95% CI, 1.485-4.166, p=0.01). During pregnancy, PLAO women showed a higher prevalence of insufficient weight gain (49.0%), calcium-intake <500 mg/day (67.1%), history of bed rest (21.9%) and use of low-molecular-weight heparin (LMWH, 25.2%). An increased risk of PLAO was associated with calcium intake <500 mg/day during pregnancy (OR 1.5, 95% CI, 1.047-2.141, p=0.027), insufficient weight gain (OR 1.8, 95% CI, 1.109-3.003 p=0.018) and LMWH use (OR 2.0, 95% CI, 1.124-3.682, p=0.019).

**Conclusions:**

PLAO, a condition with relevant impact on women’s health, is associated with BMI <18.5, low calcium intake, age at pregnancy, previous fragility fractures and the presence of chronic diseases before pregnancy and with insufficient weight gain, LMWH use and low calcium intake during pregnancy.

## Highlights

Pregnancy and lactation-associated osteoporosis (PLAO) is a rare but severe condition characterized by vertebral or other fragility fractures during late pregnancy/postpartum period. This study, based on a nationwide survey in PLAO conducted among 155 women with PLAO and 182 controls suggests that underweight, low calcium intake, age at onset of pregnancy, previous fragility fractures and the presence of chronic diseases could be associated with bone fragility are factors, assessable before pregnancy, associated with PLAO. Moreover, insufficient gestational weight gain, heparin use and reduced calcium intake represent additional risk factors to consider during pregnancy.

## Introduction

Pregnancy and lactation-associated osteoporosis (PLAO) is a rare but severe condition characterized by bone fragility, often presenting as vertebral or other fragility fractures during late pregnancy or the postpartum period. This condition has a profound impact on maternal health, quality of life, and functional independence (1).

First described by Nordin and Roper in 1955, the PLAO incidence is estimated at approximately 0.4 per 100,000 women (2). The most recent data on the incidence of vertebral and hip fragility fractures during pregnancy, puerperium, and lactation come from a Japanese study, which reports an incidence of approximately 4.6 per 10,000 women (3). However, it is widely assumed that the true incidence is underestimated (4). Severe cases, often highlighted in the literature, report alarming cascades of 5 to 10 vertebral compression fragility fractures postpartum. While these represent the hallmark of PLAO, they likely reflect a reporting bias toward the most severe cases (5).

In addition to vertebral fractures (VFXs), fragility fractures in other skeletal regions, such as the hip acetabulum, sacrum, pubic bone, humerus, and tibia, have been documented (5). Fragility fractures outside the vertebral and femoral regions may often be regarded as common or insufficiently atypical to prompt a thorough bone health evaluation, leading to potential underreporting (2). Another recognized condition during pregnancy is the transient regional osteoporosis of the hip (TOH), which can result in hip pain or fragility fractures during late pregnancy or shortly after delivery. TOH may sometimes involve bilateral hip fragility fractures and its pathophysiology is poorly understood, though ischemia or uterine pressure have been implicated (6, 7).

Physiological adaptations during pregnancy and lactation aim to meet the increased calcium and phosphate demands of fetal development and milk production. These adaptations include enhanced intestinal calcium absorption during pregnancy, mobilization of maternal skeletal stores, and increased renal calcium excretion (2). Parity and lactation are not associated with an increased risk of osteoporosis or fragility fractures in the general population. On the contrary, they may have long-term protective effects. After weaning, bone mineral density (BMD) typically recovers and some parameters such as Hip and tibia strength indexes evaluated by pQCT may even improve (8–10).

However, during the late stages of pregnancy or within the first six months postpartum, particularly during lactation, some women develop fragility fractures. Emerging evidence suggests that PLAO results from a multifactorial etiology, involving genetic predisposition, hormonal changes, nutritional deficiencies, and potentially pre-existing low BMD (11).

From a clinical point of view, women diagnosed with PLAO, are often typically apparently healthy and lack classical risk factors for postmenopausal osteoporosis (25). Consequently, prior to the index pregnancy BMD measurements are generally available in a minority of women (10). The limited epidemiological data further complicates our understanding of PLAO. Most studies are based on small case series or without control groups, leaving significant gaps in knowledge regarding its prevalence, risk factors, and optimal management strategies (11).

This survey-based case-control study aims to address these gaps by presenting the results obtained from women with PLAO and a matched control group. The primary objectives are to describe the clinical characteristics of PLAO in the Italian population, focusing on fragility fractures, diagnostic evaluations, and therapeutic approaches, and to identify potential risk factors for PLAO, both unrelated and related to pregnancy.

## Patients and methods

From December 2024 to February 2025, an anonymous online survey was distributed to all members of MAMog (Mamme con Osteoporosi Gravidica, Mothers with Pregnancy related Osteoporosis), an Italian association established in 2023 by women affected by PLAO. MAMog members were encouraged to forward the survey to one or more unrelated peers of similar age to obtain a control group. The survey was designed to collect data on clinical history, lifestyle factors, nutritional habits, and pregnancy-related characteristics.

The inclusion period was limited to this three-month window because all eligible MAMog members and their referred controls responded within this timeframe. Extending the data collection period beyond February 2025 would not have led to the inclusion of additional participants, as all active members fitting the study criteria had already been reached.

All women associated with MAMog were asked to provide information about the fragility fractures site and the severity of the trauma. The inclusion criteria were: i) the occurrence of VFXs or TOH during last trimester of pregnancy or during the first six months after delivery; ii) the occurrence of VFXs in the absence of trauma or in the presence of a trauma equivalent to a fall from standing height or less. Only women with radiologically confirmed vertebral fractures (e.g., via spine X-ray, MRI, or CT scan) or transient osteoporosis of the hip (TOH) were included. Imaging confirmation was mandatory to ensure diagnostic accuracy and to exclude self-reported but unverified cases. Women who reported other fragility fractures defined by site, timing or trauma were excluded. Fractures other than vertebral or femoral fractures were excluded due to the greater difficulty in confirming their non-traumatic nature (3 patients). Women could participate regardless of the current menopausal status, history of known (secondary) causes of bone fragility or prior osteoporosis treatment. This choice was based on the fact that the diagnosis of PLAO was strictly defined by the timing of fragility fractures in relation to pregnancy or lactation, which clearly occurred in the premenopausal or peripartum period. Therefore, current menopausal status at the time of survey completion had no impact on inclusion criteria or on the interpretation of pregnancy-related risk factors. Individual survey identifiers prevented inclusion of duplicate subject data. The survey was distributed via web-link in a confidential manner, developed using REDCap (Research Electronic Data Capture), a secure web application for building and managing online surveys and databases (https://projectredcap.org/software/). The online survey was designed to take approximately 20–30 min to complete and included multiple choice, numerical response, and text responses questions type.

The anonymous online survey was designed to gather comprehensive information on factors potentially associated with PLAO. Specifically, the questionnaire collected data in the following areas:

1. Family history: i) history of vertebral or femoral fragility fractures in first-degree relatives and presence of autoimmune diseases in first-degree relatives; ii) connective tissue symptoms: presence of blue sclerae in first-degree relatives.
2. Personal medical history and lifestyle habits: i) previous Fragility fractures, including site, number, and trauma mechanism; ii) history of active smoking (≥5 cigarettes/day) or past smoking; iii) use of glucocorticoids before or during pregnancy; iv) history of kidney stones, amenorrhea, eating disorders, hearing loss, dental issues, such as frequent cavities or enamel problems; v) known chronic diseases; vi) connective tissue symptoms: presence of abnormal scarring (e.g., widened scars or “cigarette-paper” scars) or blue sclerae, or possible hypermobility evaluated by a five-part questionnaire “5PQ” (a score of 2 or higher is considered to have the best compromise between sensitivity and specificity (13). This test was also validated in pregnant women and a score ≥2 was identified to rule-out women without generalized joint hypermobility (14). To be more specific, we arbitrarily chose a cutoff of 3 to define the presence of suspected hypermobility); vii) structured physical activity at least once a week before pregnancy (during adulthood). We categorized participants as physically active if they reported engaging in at least one hour per week of structured physical activity, which is broadly consistent with the WHO 2020 recommendations for vigorous-intensity aerobic activity (≥75 minutes/week) (15); viii) weight, height, body mass index (BMI) before pregnancy: women were categorized as underweight, normal weight, overweight and obese (BMI<18.5, 18.5-24.9, 25.0-29.9, ≥30.0 Kg/m^2^, respectively); ix) skin phototype: all women were asked to define their skin phototype according to the Fitzpatrick classification, based on a representative image of the six standard skin phototypes uploaded in the survey”.
3. Reproductive and Pregnancy-Related Factors: i) use of assisted reproductive technologies; ii) dietary calcium intake during pregnancy, expressed as mg/day and assessed using a simplified questionnaire (16) the calcium intake, was classified as “ substantially adequate” (daily calcium intake of ≥1000 mg/day, consistent with the recommended levels for pregnancy), “ inadequate” (daily calcium intake between 500 mg/day and 999 mg/day, reflecting insufficient but not critically low levels), or “severely inadequate” (daily calcium intake of <500 mg/day, representing a level likely insufficient to support maternal and fetal calcium needs despite compensatory mechanisms) (1); iii) use of vitamin D supplementation during pregnancy; iv) history of bed rest ≥30 days (17); v) use of heparin during pregnancy (subjects were not asked to specify the type of heparin because only low molecular weight heparin is used in Italy in pregnancy); vi) history of hypertension or diabetes mellitus developed during pregnancy; vii) gestational weight gain: since the the weight gain considered adequate is 12.5–18.0 kg in underweight, 11.5–16.0 kg in normal weight, 7.0–11.5 kg in overweight, and 5.0–9.0 kg in obese wome (18), women were categorized as with adequate or insufficient or excessive weight gain if the weight gain fell within or below or above the recommended range based on their pre-pregnancy BMI, respectively; viii) gestational age at delivery; ix) month of delivery; x) type of delivery (vaginal or cesarean).

Only in PLAO women, we also investigated: i) the timing of symptoms onset in relation to pregnancy or postpartum; ii) the time interval between the onset of symptoms and the PLAO diagnosis; iii) the diagnostic tests performed to evaluate skeletal metabolism (e.g. Dual X-ray densitometry - DXA, biochemical exams, specific exams for excluding secondary osteoporosis, genetic analysis); iv) if a diagnosis of secondary osteoporosis, in addition to PLAO, was established; v) if they had changed their dietary calcium intake after diagnosis; vi) if they had introduced vitamin D or calcium supplementation if not previously used; vii) if they had undergone vertebroplasty; viii) if they started taking osteoporosis medication, specifying the type of medication used (e.g., bisphosphonates, teriparatide, or other osteoporosis treatments).

We have received authorization from both the Director of the Hospital Facility and the Medical Director (13/11/2024, Milan) to be exempt from the requirement to submit our study to the Ethics Committee and to obtain informed consent. This exemption was granted as we will be using an anonymous questionnaire with no clinical objectives.

### Statistical analysis

A thorough data analysis was conducted to identify and address any potential inconsistencies among women and controls response. Statistical analyses were performed using SPSS (IBM Corp., Version 29, Armonk, NY). The results were expressed as mean ± SD or median (IQR). The normality of distribution was tested by Kolmogorov–Smirnov test. The comparison of continuous variables was performed using Student’s *t*-test or Mann–Whitney U-test, as appropriate. Categorical variables were compared by χ^2^ test or Fisher’s exact test, as appropriate. Results were considered significant with p <0.05.

We decided to use logistic regression to evaluate the pregnancy-unrelated and pregnancy- related risk factors associated with PLAO. Given the high number of variables showing significant differences between PLAO and controls, we included only those with the most significant differences (i.e., p ≤ 0.02) in the analysis. We tested the following potential risk factor associated with PLAO: i) pregnancy-unrelated risk factors: family history of vertebral or hip Fragility fractures, pre-pregnancy Fragility fractures, chronic diseases associated with skeletal fragility, blue sclerae, age at pregnancy, BMI <18.5 Kg/m2, daily calcium intake <500 mg/day; ii) pregnancy-related risk factors: BMI <18.5 Kg/m2, daily calcium intake <500 mg/day during pregnancy, insufficient weight gain during pregnancy, use of heparin during pregnancy.

No formal sample size calculation was performed *a priori* due to the exploratory nature of the study and the rarity of PLAO. All eligible and available subjects were included to maximize statistical power.

## Results

The survey was sent to all MAMog members (n = 175). Of these, 158 responded, among whom 3 were excluded due to not meeting the inclusion criteria for fragility fractures. One-hundred-eighty-two women without PLAO responded and were included as controls. Overall, 337 women completed the survey: 155 with PLAO and 182 without PLAO, who served as controls.

### Description of PLAO group

#### Clinical characteristics of patients

Among the 155 women with PLAO, 148 (95.5%) reported VFXs and 7 TOH (4.5%). In 78.1% of women fragility fractures occurred at their first (78.4% VFXs and 71.4% TOH), 16.8% at their second (16.2% VFXs vs 28.6% TOH), and 5.2% at their third or fourth (5.5% VFXs vs 0.0% TOH) pregnancy, respectively. The mean number of VFXs per patient was 5.0 ± 2.7 (range 1-13). Among women with TOH, 42.9% and 57.1% had unilateral and bilateral involvement, respectively.

Among women with VFXs the pain appeared in the last weeks of pregnancy, immediately after delivery, in the first 3 months post-partum and between 3- and 6-months post-partum in 22.3%, 18.1%, 51.0% and 5.4% of women, respectively. In all women with TOH pain appeared before delivery.

The diagnosis of VFXs or TOH was made immediately in 7.1% of cases (6.8% and 14.3% in women with VFXs and TOH, respectively, p=0.448), within 1 month after pain onset in 16.1% of cases (14.8% and 1.3% in women with VFXs and TOH, respectively, p=0.360), 1–2 months after pain onset in 32.9% (32.4% and 42.9% in women with VFXs and TOH, respectively, p=0.566) and more than 2 months after pain onset in 43.9% (45.3% and 14.3% in women with VFXs and TOH, respectively, p=0.106).

Among women with VFXs who sustained the fracture after childbirth 81.9% (95 out of 116) were exclusively breastfeeding, 13.8% were practicing mixed feeding (16 out of 116), and 4.3% (5 out of 116) were not breastfeeding. Among these subjects, after diagnosis, the 27.9% did not discontinue breastfeeding, 45.0% discontinued breastfeeding within 1 month, 27.0% gradually discontinued breastfeeding within 2 months, with no difference among women who had exclusive breastfeeding or mixed breastfeeding (data not shown).

#### Diagnostic procedures and secondary osteoporosis diagnosis

A DXA evaluation and generic biochemical tests for osteoporosis were performed in 93.5% and 94.8% of cases, respectively. More extensive tests for secondary osteoporosis screening were performed in 49.7% of cases and, eventually, genetic analysis to rule out monogenic forms of osteoporosis in 20.0% of cases.

Among the 31 women undergoing genetic analysis, a positive result was found in 7 cases (22.6%), thus at least 4.5% of the whole PLAO population was affected by a genetic form of bone fragility (osteogenesis imperfecta (OI), Ehlers Danlos Syndrome (EDS), OI/EDS and Monogenic Juvenile Osteoporosis due to PLS3 mutation were diagnosed in 4, 1, 1 and 1 women, respectively).

Analyzing data on previously non-recognized clinical manifestations of collagenopathy (marked hypermobility score ≥3, blue sclerae, precipitous delivery, and slashed scars) and considering the presence of ≥2 criteria or the isolated presence of a hypermobility score ≥4 or blue sclerae, we identified 7 women with clinical features possibly indicative of collagenopathy: 2 women were identified among those in whom another secondary cause of bone fragility was identified, 4 cases were identified in women who had not undergone genetic analysis, and 1 case was suspected in a woman in whom genetics was negative (hypermobility score 5 and slashed scars compatible with a possible hypermobile Ehlers-Danlos syndrome, for which, unlike other types, the causative gene remains unknown) (19). If these 7 cases were confirmed, the proportion of women with confirmed or suspected collagenopathies/monogenic osteoporosis within our cohort would reach 9.0%. This figure does not represent a population-level prevalence estimate but rather an observation based on the available sample.

In 3 women (1.9%) a newly diagnosed possible cause of PLAO (primary hyperparathyroidism, systemic indolent mastocytosis and Graves-Basedow disease) was found at secondary osteoporosis screening test other than genetic one. In additional 22 women (14.2%) an already known disease could explain a higher risk for fragility fractures. In particular, 11 women were affected with gastroenterological disease (ulcerative or other kind of colitis, coeliac disease and gastrointestinal stromal tumor). The remaining 11 women were already known to be affected by undifferentiated connective tissue disease, Bechet disease, Graves Basedow disease, rheumatoid arthritis, multiple sclerosis and Anorexia Nervosa, in 2, 1, 1, 3, 3, 1 cases, respectively. To summarize, overall, a cause of secondary bone fragility was present in 20.6% of PLAO.

No difference was found in the number of vertebral fractures between patients with and without secondary osteoporosis (data not shown).

#### Therapy and management of patients with PLAO

Only 16.8% of women took vitamin D during pregnancy. After the diagnosis of fragility fractures this percentage increased to 95.5%. The 61.3% of women with PLAO started taking calcium supplements and 78.1% reported trying to increase dietary calcium intake. After diagnosis of fragility fractures, 101 women (65.2%) introduced osteoporosis medications: teriparatide in 42 women, 31 underwent therapy for at least 18 months, while the others received treatment for a maximum of 6 months, bisphosphonates in 52 women, in approximately half of the cases for less than 12 months, denosumab in 5 women, romosozumab in 1 patient, a therapeutic sequence in 12 women. 85.2% of cases continued to have specialist visits. Most bisphosphonates were administered orally, although a minority of women received intramuscular formulations, such as neridronate or clodronate. Among the women treated with teriparatide, some reported using it for only 6 months. Although specific reasons were not collected, this may reflect practical limitations such as lack of reimbursement or financial constraints. Overall, the reported treatments were not standardized and reflect the heterogeneity of therapeutic approaches adopted across different clinical settings, rather than study-driven decisions. The percentage of women with a follow-up was significantly lower in women with TOH (57.1%) than in those with VFXs (86.5%, p=0.033).

### Comparison between women with PLAO and controls: pre-pregnancy risk factors

The clinical, pre-pregnancy, characteristics of PLAO and controls are reported in **Table 1**.

**Table 1:** Clinical pre-pregnancy characteristics of PLAO and controls.

Variable	All (n=337)	PLAO (n=155)	Controls (n=182)	P
Age at delivery (years)	32.2±5.1	32.9±4.7	31.6±5.4	0.012
Weight (Kg)	58.1±9.7	56.0±8.9	59.9±10.0	0.0001
Height (meter)	1.65±0.06	1.65±0.06	1.65±0.06	0.421
BMI (Kg/m^2^)	21.3±3.2	20.5±2.7	21.9±3.4	0.0001
BMI <18.5/18.5-24.9/25-29.9/≥30 (%)	18.5/69.9/9.2/2.3	23.6/70.1/5.5/0.7	13.8/69.8/12.5/3.8	0.005
BMI <18.5 Kg/m^2^ (%)	18.5	23.6	13.8	0.002
History of eating disorders (%)	8.3	11.6	5.5	0.049
History of amenorrhea (%)	4.4	7.1	2.2	0.035
Chronic diseases associated with skeletal fragility (%)	6.8	11.7	2.6	0.002
Prevalent fragility fractures (%)	11.8	18.7	6.0	0.001
Prevalent fragility fractures: 0/1/≥2 (%)	87.0/7.7/5.3	79.4/11.0/9.7	93.4/4.9/1.6	0.001
Daily calcium intake ≥1000 /500-999 /<500 mg/day (%)	9.5/32.3/58.2	7.1/26.0/66.9	11.5/37.7/50.8	0.012
Glucocorticoid use ≥3 months (%)	5.6	4.5	6.6	0.411
Blue Sclerae	2.7	5.2	0.5	0.009
Abnormal scarring if assessable (%)	11.7	16.2	8.2	0.054
History of anaphylactic shock (%)	2.4	1.3	3.3	0.296
History of allergy (%)	4.3	3.9	4.5	0.792
Familial History of vertebral or hip fragility fractures (%)	16.0	21.0	11.0	0.005
Regular structured physical activity (%)	53.3	47.7	57.9	0.064
History of kidney stones (%)	23.1	24.5	21.9	0.605
Number of dental issues	0.6±0.8	0.6±0.8	0.6±0.8	0.963
Periodontal disease (%)	5.3	4.5	6.0	0.631
Dental enamel alterations (%)	6.2	6.5	6.0	1.000
Tooth decay (≥1) (%)	37.1	38.1	36.3	0.736
Dental Abscesses (≥1) (%)	11.9	11.6	12.1	1.000
Tooth loss (≥1) (%)	3.6	3.9	3.3	0.777
Hypermobility score: 0-1/2-3/4-5	75.4/18.7/5.9	77.4/18.7/3.9	73.6/18.7/7.7	0.330
Hearing loss (%)	3.0	3.2	2.7	0.790
Active smoke (≥5 cigarettes/day) (%)	9.2	11.0	7.7	0.346

Data are expressed as mean±SD if not differently specified or percentage in parentheses for continuous and categorical variables respectively.

BMI, body mass index.

Women with PLAO were slightly older, with lower weight and BMI and a higher prevalence of body underweight (BMI <18.5). Moreover, they also presented a higher prevalence of eating disorders and amenorrhea and chronic diseases known to be possibly associated with skeletal fragility.

They also had a higher prevalence of pre-pregnancy fragility fractures, either considered as a dichotomous variable or considering the presence of one or more fragility fractures. They also have a higher prevalence of vertebral or femoral fragility fractures in first-degree relatives. Daily calcium intake before pregnancy was more frequently inadequate or severely inadequate in women with PLAO than in controls. Similarly, in PLAO subjects the presence of blue sclerae was higher (p=0.009) and of abnormal scarring tended to be more frequent (p=0.059) as compared with controls, while a structured physical activity before pregnancy tended to be less frequent in PLAO women than in controls (p=0.064).

No differences were found in the chronic use of glucocorticoids, history of kidney stones, active smoking, hearing loss, anaphylactic shock or allergy, dental problems, score for suspected hypermobility and frequency of different photo-types (p=0.331). Analyzing the data after excluding women with TOH, the results were confirmed (data not shown).

**Figure 1:**
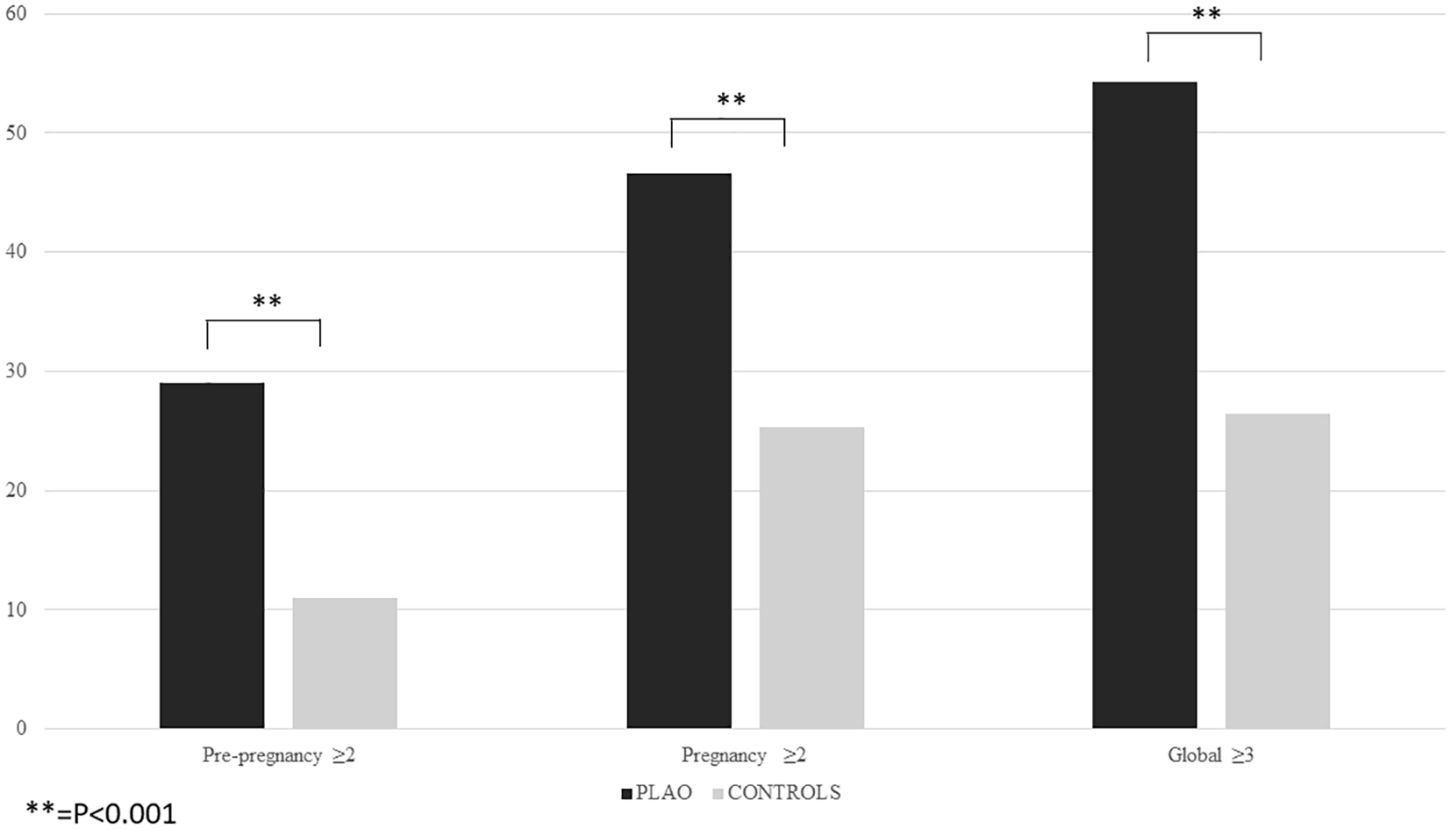
Risk factors for PLAO.

The logistic regression analysis revealed that the presence of pre-pregnancy fragility fractures, chronic diseases associated with skeletal fragility, age at pregnancy, body underweight and a daily calcium intake <500 mg/day were independently associated with an increased risk of PLAO (**Table 2**). The 29% of women with PLAO had at least two of these factors, compared to 11% of the control (p=0.001) (**Figure 1**). The presence of at least two out of four of these factors, excluding age, has a sensitivity of 29.0%, a specificity of 89.0% and a positive predictive value of 69.2% for PLAO.

**Table 2:** Logistic regression for possible pre-pregnancy contributors to PLAO.

	OR	95% CI	P
Family history of vertebral or hip fragility fracture (presence)	1.9	0.966-3.712	0.063
Prevalent fragility fracture (presence)	2.7	1.199-6.036	0.016
Chronic diseases associated with skeletal fragility (presence)	5.8	1.919-17.562	0.002
Blue sclerae (presence)	7.0	0.734-66.464	0.091
Pregnancy age (1-year increase)	1.1	1.005-1.109	0.030
BMI <18.5 Kg/m^2^ (presence)	2.0	1.042-3.902	0.037
Daily calcium intake <500 mg/day (presence)	2.5	1.485-4.166	0.001

OR: odds ratio; 95% CI, 95% interval of confidence.

### Comparison between women with PLAO and controls: pregnancy risk factors

The clinical characteristics of PLAO and controls, during pregnancy, are reported in **Table 3**.

**Table 3:** Clinical characteristics of PLAO and controls during pregnancy.

	All (n=337)	PLAO (n=155)	Controls (n=182)	P
Age at delivery (years)	32.2±5.1	32.9±4.7	31.6±5.4	0.012
BMI <18.5 Kg/m^2^ (%)	18.5	23.6	13.8	0.002
Gestational WG (Kg)	12.7±4.5	11.9±4.0	13.3±4.7	0.005
Insufficient WG (%)	39.6	49.0	31.4	0.001
Excessive WG (%)	20.6	16.7	24.1	0.127
Daily calcium intake <500 mg/day (%)	58.2	67.1	50.5	0.003
Taking vitamin D supplements, in addition to multivitamins (%)	16.3	16.8	15.9	0.835
Glucocorticoid use ≥3 months (%)	5.6	6.5	3.8	0.322
Development of hypertension (%)	3.9	1.3	6.0	0.025
Development of gestational diabetes (%)	5.9	3.9	7.7	0.168
Use of low molecular weight heparin (%)	19.8	25.2	15.3	0.028
History of bed rest ≥1 month (%)	17.2	21.9	13.1	0.042
Active smoke (≥5 cigarettes/day) (%)	9.2	11.0	7.7	0.346
Use of assisted reproductive technologies (%)	10.4	12.9	8.2	0.209
Gestational age at delivery (weeks)	39.0±1.5	38.9±1.6	39.1±1.5	0.263
Regular vaginal delivery	45.3	37.9	51.4	0.016
Precipitous delivery	5.0	5.9	4.3	0.619
Natural childbirth with use of forceps or suction cup	14.5	14.4	14.6	1.000
Urgent cesarean	14.2	17.0	11.9	0.211
Scheduled cesarean	21.5	25.3	18.2	0.139

Data are expressed as mean±SD if not differently specified or percentage in parentheses for continuous and categorical variables respectively.

BMI, body mass index; WG, weight gain.

Women with PLAO had a higher prevalence of body underweight at the beginning of pregnancy and of an insufficient weight gain during pregnancy. During pregnancy, as compared with controls, in women with PLAO a severely inadequate daily calcium intake, the history of bed rest ≥30 days and the use of heparin were more frequent, while vaginal delivery, gestational diabetes and hypertension development during pregnancy were less frequent. No differences were found between PLOs and controls in the intake of vitamin D supplements during pregnancy (in addition to multivitamins, daily dose of cholecalciferol ranging between 1,000 and 2,000 IU), chronic use of glucocorticoids during pregnancy, active smoking during pregnancy, use of assisted reproductive technologies and the frequency of delivery in specific months of the year (p=0.641). Analyzing the data after excluding women with TOH did not change the results (data not shown).

The logistic regression analysis revealed that, during pregnancy, a daily calcium intake <500 mg/day, an insufficient weight gain and the use of heparin during pregnancy were independently associated with an increased risk of PLAO (**Table 4**).

**Table 4:** Logistic regression for possible pregnancy contributors to PLAO.

	OR	95% CI	P
BMI below 18.5 Kg/m^2^ (presence)	1.68	0.894-3.177	0.107
Daily calcium intake <500 mg/day during pregnancy (presence)	1.50	1.047-2.141	0.027
Insufficient weight gain during pregnancy (presence)	1.82	1.109-3.003	0.018
Use of low molecular weight heparin during pregnancy	2.03	1.124-3.682	0.019

OR, odds ratio; 95% CI, 95% interval of confidence.

46.4% of women with PLAO had at least two of these factors, compared to 25.3% of the controls (p=0.001) (**Figure 1**). The presence of at least two out of four of these factors, excluding age, has a sensitivity of 46.4%, a specificity of 74.7% and a positive predictive value of 61.0%.

Moreover, 54.2% of women with PLAO had at least three factors out of the ones associated with PLAO before and during pregnancy, compared to 26.4% of the control (p=0.001) (**Figure 1**). The presence of at least two out of four of these factors, excluding age, has a sensitivity of 54.2%, a specificity of 73.6% and a positive predictive value of 63.6%.

## Discussion

The present study is the second largest investigation on PLAO (20) and the largest one among those with a control group (1, 12, 21, 22). Our data confirm that VFXs are much more frequent than TOH (95.5%) and, importantly, highlight the severity of this condition, evidenced by the high number of VFXs per patient (mean 5.6) (1, 12, 20, 22).

The observed fragility fractures timing (i.e. generally occurring in the last trimester of pregnancy for TOH and within the first three months postpartum for VFXs) as well as the association with breastfeeding, align with previous literature findings (1, 12, 20–24). As an example of a population at high risk for fractures, women with osteogenesis imperfecta (OI) represent a valuable model to explore the potential impact of breastfeeding on postpartum fracture risk, a previous study conducted on women with OI found that breastfeeding was more frequent in fractured women (85.7%) than in non−fractured ones (47.1%) (24), with all women experiencing post−partum fractures being breastfeeding, thus suggesting the importance of advising against breastfeeding in women with OI.

The management of PLAO is highly heterogeneous in Italy. Among women who sustained fragility fractures during breastfeeding, 27.9% did not discontinue breastfeeding, despite it is a well-known risk factor for fragility fractures (2, 24), as it leads to a marked increase in bone resorption, while its cessation is associated with a reduction in bone resorption and a recovery of bone mass (25). While DXA is performed in nearly all women with PLAO, second-level screening tests and genetic analyses are conducted in fewer than 50% and 20% of cases, respectively. This is despite the well-established need to rule out secondary causes of fragility fractures (26) and the recent data indicating that up to 50% of women with PLAO may harbor pathogenic mutations in genes implicated in bone fragility, such as *LRP5, WNT1*, and *COL1A1/A2* (27).

Although spontaneous BMD recovery after weaning is well documented (10-70%) (1, 8), the efficacy of osteoporosis medications is still debated in PLAO (26, 28, 29) and the use of antiresorptive drugs, could potentially blunt post-weaning recovery in a period during which the bone loss process—driven by PTH-related protein (PTHrP) produced by the lactating mammary gland—naturally comes to an end (26, 28). In our study, approximately two-thirds of women received a pharmacological treatment immediately after diagnosis: in half of these cases, bisphosphonates were prescribed and in one third teriparatide. Only a minority received denosumab or romosozumab. Current expert recommendations suggest that pharmacological treatment should be considered only after evaluating the extent of spontaneous BMD recovery 12–18 months post-weaning, if the Z score is still below expected for age (26, 29).

This recommendation is based on international guidelines, which advise the use of Z-scores (instead of T-scores) in premenopausal women, as they provide a more appropriate age-matched reference for evaluating bone mass in younger populations.

When analyzing pre-pregnancy risk factors, we identified several differences between women with PLAO and controls: prevalent fragility fractures, older age, lower BMI, body underweight, history of eating disorders, amenorrhea, skeletal fragility associated chronic diseases, familiar history of fragility fracture, inadequate calcium intake and presence of blue sclerae, which may suggest a possible underlying collagenopathy. However, in particular, we found that the presence of pre-pregnancy fragility fractures, skeletal fragility associated chronic diseases, body underweight, severely inadequate daily calcium intake and the age at pregnancy were the independent predictors of PLAO. Importantly, although previously hypothesized as potential contributors of PLAO (30, 31), these factors had never been clearly confirmed in a single controlled study, so far. Indeed, the current lack of knowledge of all potential risk factors for PLAO is due to the fact that the four studies in the literature including women with PLAO and a control group (1, 12, 21, 22) were small and did not explore all potential risk factors for PLAO.

Interestingly, previous studies have reported a reduced physical activity in women with PLAO (1, 12, 21). In keeping with these data, even in our study in women with PLAO a regular and structured physical activity prior to pregnancy tended to be less frequent than in controls, thus confirming the role of physical activity in youth as a determinant of peak BMD attainment and, ultimately, on the risk of PLAO.

Moreover, unlike previous studies (12), which excluded women with pre-pregnancy conditions potentially associated with secondary osteoporosis, our study demonstrated that the presence of chronic diseases known to impact skeletal fragility is a significant risk factor for PLAO. However, to date, the awareness of the potential bone involvement in young individuals with chronic diseases remains low, and in most cases, no preventive strategies are implemented to preserve bone strength in this population, nor during childbearing age in anticipation of a possible pregnancy.

When analyzing pregnancy related risk factors, the present study showed that during pregnancy in women with PLAO the frequency of the need of bed rest (≥1 month) was higher than in controls, and, importantly that the presence of a severely inadequate daily calcium intake, an insufficient weight gain and the use of low molecular weight heparin were independently associated with an increased risk of PLAO. Our findings are in keeping with those of previous controlled studies (12, 21, 22), which reported a higher prevalence of immobilization (≥1 month) and use of low weight heparin during pregnancy in PLAO women.

Although prior reviews, such as the one by Ginsberg et al. (33), concluded that low-molecular-weight heparins (LMWH) do not significantly increase the risk of osteoporosis in pregnant women, several case reports and small case series of PLAO have previously noted the use of LMWH in affected individuals (12, 22, 23). Our study is the first to document this association in a large case-control cohort and to demonstrate its statistical significance. While LMWH alone is unlikely to cause osteoporosis in otherwise healthy pregnant women, it may contribute to PLAO when combined with other risk factors such as undernutrition, low calcium intake, or immobilization. Additionally, LMWH use may reflect the presence of obstetric complications (e.g., thrombophilia, prolonged bed rest), which themselves may compromise bone health. These findings support a multifactorial pathophysiological model, where LMWH serves as a possible contributing factor rather than a primary cause.

Differently from what was reported by Hadji (12), we found lower prevalence of pregnancy hypertension in PLAO women, possibly related to the fact that our women with PLAO had more frequently body underweight and insufficient weight gain during pregnancy, since it is well known that a high BMI is a risk factor for preeclampsia. From a clinical point of view the entirely new finding that an insufficient weight gain during pregnancy could represent a risk factor for PLAO is of importance. Indeed, it is conceivable that an inadequate weight gain during pregnancy may represent, as well as low BMI, previous eating disorder and low calcium intake, a sign of suboptimal nutrition that can contribute to osteomalacia, which probably represents a very important feature for PLAO.

The main limitation of this study is its retrospective design and the reliance on self-reported data, which may introduce recall or reporting bias. However, the same standardized anonymous survey was administered to both cases and controls, reducing the likelihood of differential bias. In addition, rigorous data cleaning was performed to minimize inconsistencies. While causality cannot be inferred, the strength and consistency of associations observed, together with their biological plausibility, support the relevance of the identified risk factors. Nonetheless, our conclusions are framed in terms of associations, and we emphasize the need for prospective studies to confirm these findings. Another limitation is that the genetic analyses have been performed in a subgroup of women. However, the finding that about 23% of screened women were affected by a monogenic form of bone fragility confirms the importance of the clinical evaluation for individuating women at risk of PLAO. Finally, we have no information on subsequent pregnancies and the potential recurrence of fractures.

Notwithstanding these limitations, the present data highlight how PLAO, despite its dramatic impact, remains poorly understood, with a significant heterogeneity in both diagnostic and therapeutic approaches, likely due to the scarcity of the so far available data. The strength of the present study lies in the large sample size and the inclusion of a control group, which allowed us to suggest that women with fragility fractures, low BMI, and inadequate calcium intake prior to pregnancy should be considered at risk of PLAO. This awareness could contribute to reduce the effect of modifiable risk factors and to provide appropriate monitoring during pregnancy. In addition, the present study strongly suggests that attention should be given during pregnancy to women who present an insufficient weight gain, an inadequate calcium intake and the use of heparin. It is conceivable that in all pregnant women, calcium intake and vitamin D levels should be normalized, specific dietary recommendations should be provided to ensure adequate weight gain and, in specific cases, the option of formula feeding should be suggested before the deliver and breastfeeding discontinuation should be recommended in women with PLAO diagnosis.

In addition, the present data suggest that the diagnosis of PLAO should not be considered as the final diagnosis. The secondary causes of osteoporosis must always be investigated even in otherwise asymptomatic women (32). In particular, in the absence of an already known chronic disease potentially explaining a higher risk for fragility fractures, the second-level screening tests and genetic analyses should be considered mandatory. Women with PLAO should be referred to specialized centers for proper diagnostic and therapeutic assessment, as well as for subsequent follow-up and counseling regarding potential future pregnancies.

## Data Availability

The datasets presented in this study can be found in online repositories. The names of the repository/repositories and accession number(s) can be found below: The data is available upon request.
